# Supplementary figures and images for: Core circadian clock transcription factor BMAL1 regulates mammary epithelial cell growth, differentiation, and milk component synthesis
Source: PLoS One. 2021 Aug 20;16(8):e0248199. doi: 10.1371/journal.pone.0248199 (PMC8378744; doi:10.1371/journal.pone.0248199)

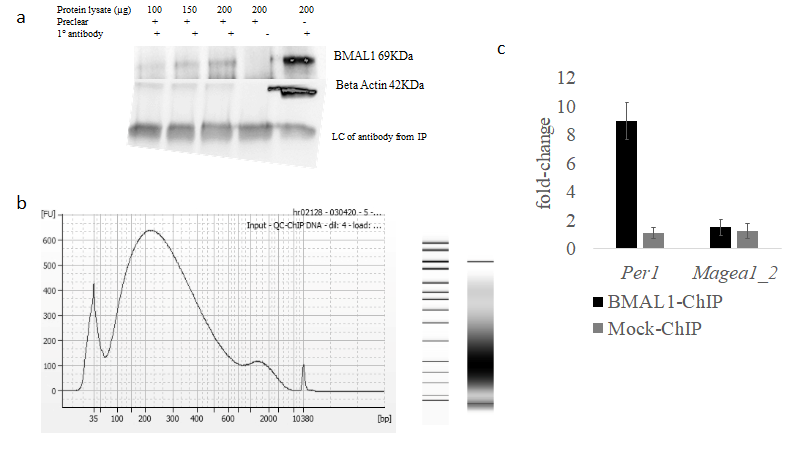

Supplement: S1 Fig — (a) Western blot analysis of immunoprecipitation (IP) of BMAL1 protein in HC11 cell protein lysate using rabbit polyclonal antibody to BMAL1 (ab3350, ChIP grade, 2 μg per IP). Lane 1 = Precision Plus Protein Standard Ladder; Lane 2 = 100 μg, Lane 3 = 150 μg, Lane 4 = 200 μg, Lane 5 = 200 μg of lysate precleared and incubated with beads but no Ab (-), Lane 6 = an aliquot of supernatant pulled off of lane 5 sample before wash steps that precede elution. western blot was performed using the mouse monoclonal antibody to BMAL 1 (sc-373955 @ 1:750 primary antibody concentration) for visualization. (b) Electropherogram analysis of input DNA used for ChIP-seq shows ideal size for next generation sequencing (seq). (c) Evaluation of antibody specificity indicated no difference between mock-ChIP and BMAL1-ChIP samples in the cycle threshold values following RT-qPCR analysis of an exon region of the Magea1_2 sperm specific gene, which is not a BMAL1:CLOCK target. Whereas a 9-fold difference in enrichment was found between RT-qPCR analysis of BMAL1-ChIP and mock-ChIP for the Per1 promoter region versus the exon region of Magea1_2. A positive ChIP was defined as at least 2-fold greater than mock-IP sample. Different letters indicate significant difference at p<0.05. (TIF) [file pone.0248199.s001.tif]

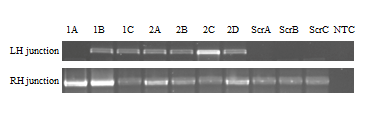

Supplement: S3 Fig — (a) PCR Analysis of monoclonal donor cassette integration. The first 10 lanes contain DNA from monoclonal colonies post clonal selection. The first character of each name refers to the gRNA used (i.e. 1A is HC11 that has undergone integration using gRNA 1). NTC denotes a no-template negative control. These results indicate proper integration of the donor cassette into the target site of cell colonies 1B, 1C, 2A, 2B, 2C, and 2D. (TIF) [file pone.0248199.s003.tif]

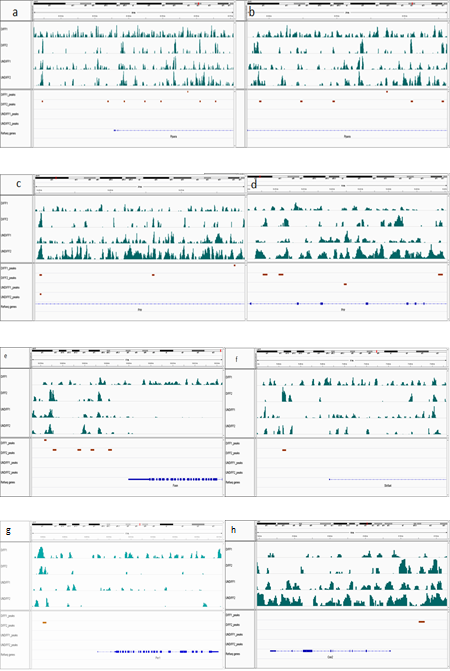

Supplement: S4 Fig — Reads per sample of peaks closest to: Ppara zoomed out (a) and in (b); Prlr (c) and (d); Fasn (e); Slc6a4 (f); Per1 (g); and Csn2 (h) transcriptional start sites. (TIF) [file pone.0248199.s004.tif]

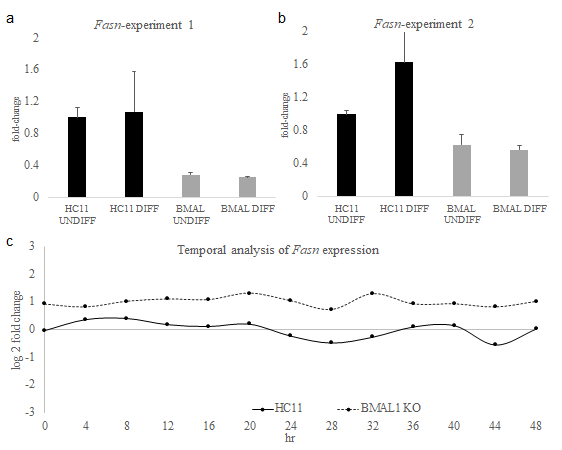

Supplement: S5 Fig — Values are mean technical replicates within experiment, normalized to express fold-change relative to mean of HC11 ± standard deviation using delta-delta cycle threshold method. Temporal analysis of Fasn expression in WT HC11 (solid line) and BMAL1-KO (dashed line) cultures. For this experiment cells were grown to confluence in growth media. Media was changed to lactogen media for 2 hr to synchronize clocks. At completion of 2 hr lactogen treatment (time 0 hr), cells were rinsed with PBS and cultured in growth media for remainder of the experiment. Cells were collected for isolation of total RNA every 4 hr over a 48 hr period beginning at 0 hr. Fasn was measured with RT-qPCR, and levels were expressed relative to mean ΔCTof HC11 across all time points. Cosinor analysis found mesor (0.01 and 0.97), amplitude (0.21 and 0.12), acrophase (-9.14 and -4.44), R2 (0.26 and 0.19) and p-value (0.22 and 0.33) of fit to a 24 hr rhythm, respectively, for HC11 and BMAL1-KO lines. (TIF) [file pone.0248199.s005.tif]
