# Supplementary material for: Core circadian clock transcription factor BMAL1 regulates mammary epithelial cell growth, differentiation, and milk component synthesis
Source: PLoS One. 2021 Aug 20;16(8):e0248199. doi: 10.1371/journal.pone.0248199 (PMC8378744; doi:10.1371/journal.pone.0248199)

Title: Core circadian clock transcription factor BMAL1 regulates mammary epithelial cell growth, differentiation, and milk component synthesis.

Authors: Theresa Casey[1‡], Aridany Suarez-Trujillo[1], Shelby Cummings[1], Katelyn Huff[1], Jennifer Crodian[1], Ketaki Bhide[2], Clare Aduwari[1], Kelsey Teeple[1], Avi Shamay[3], Sameer J. Mabjeesh[4], Phillip San Miguel[5], Jyothi Thimmapuram[2], and Karen Plaut[1]

Affiliations: 1. Department of Animal Science, Purdue University, West Lafayette, IN, USA; 2. Bioinformatics Core, Purdue University; 3. Animal Science Institute, Agriculture Research Origination, The Volcani Center, Rishon Letsiyon, Israel. 4. Department of Animal Sciences, The Robert H. Smith Faculty of Agriculture, Food, and Environment, The Hebrew University of Jerusalem, Rehovot, Israel. 5. Genomics Core, Purdue University

Supplemental material contents.

Page 2: Whole gel presented in Figure 3A.

Page 3: Whole Components Supplemental Figure S1A.

Page 4: Whole gels of supplemental Figure S2.

Blots presented in Figure 3A

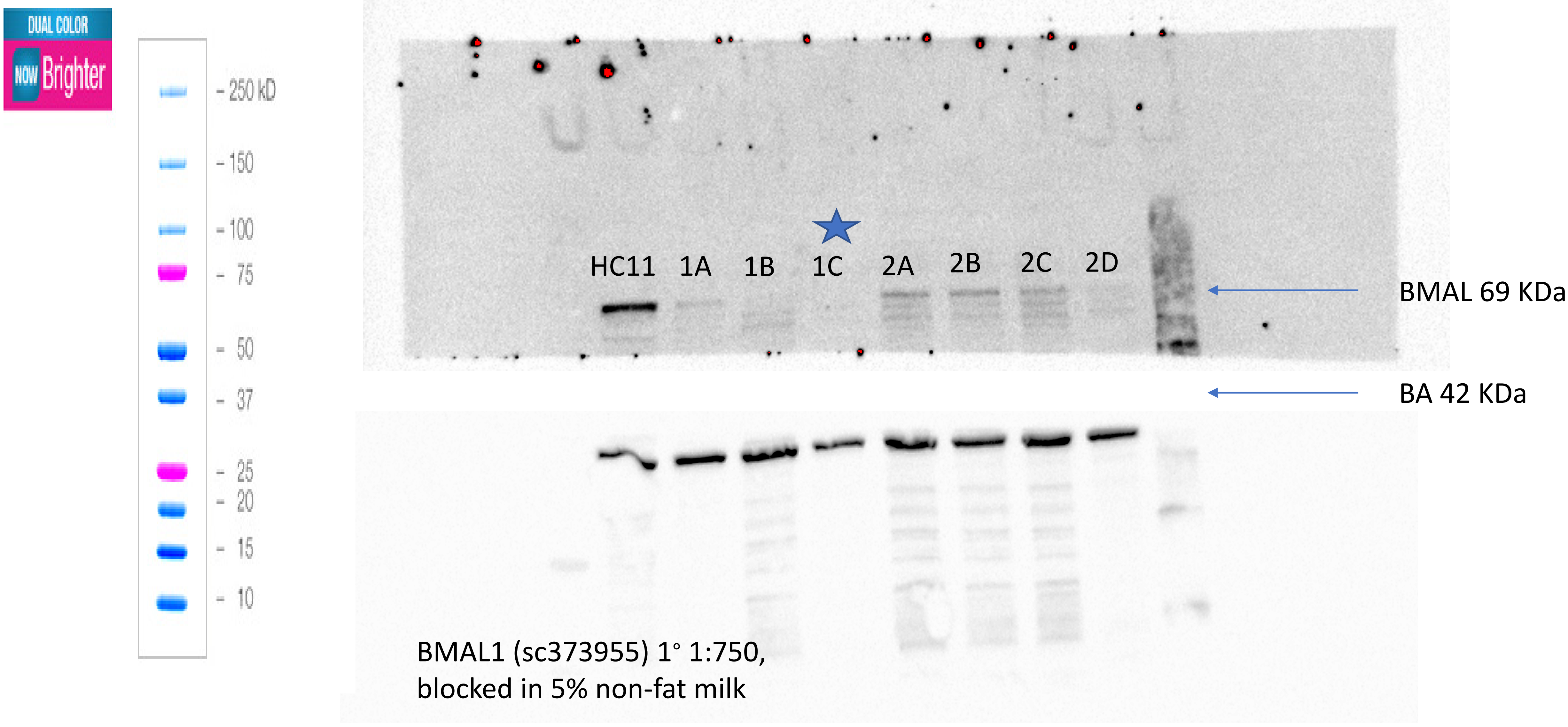

100ug samples

BA (ab8227) 1° 1:5000,
blocked in BSA

Whole Components Supplemental Figure. S1A

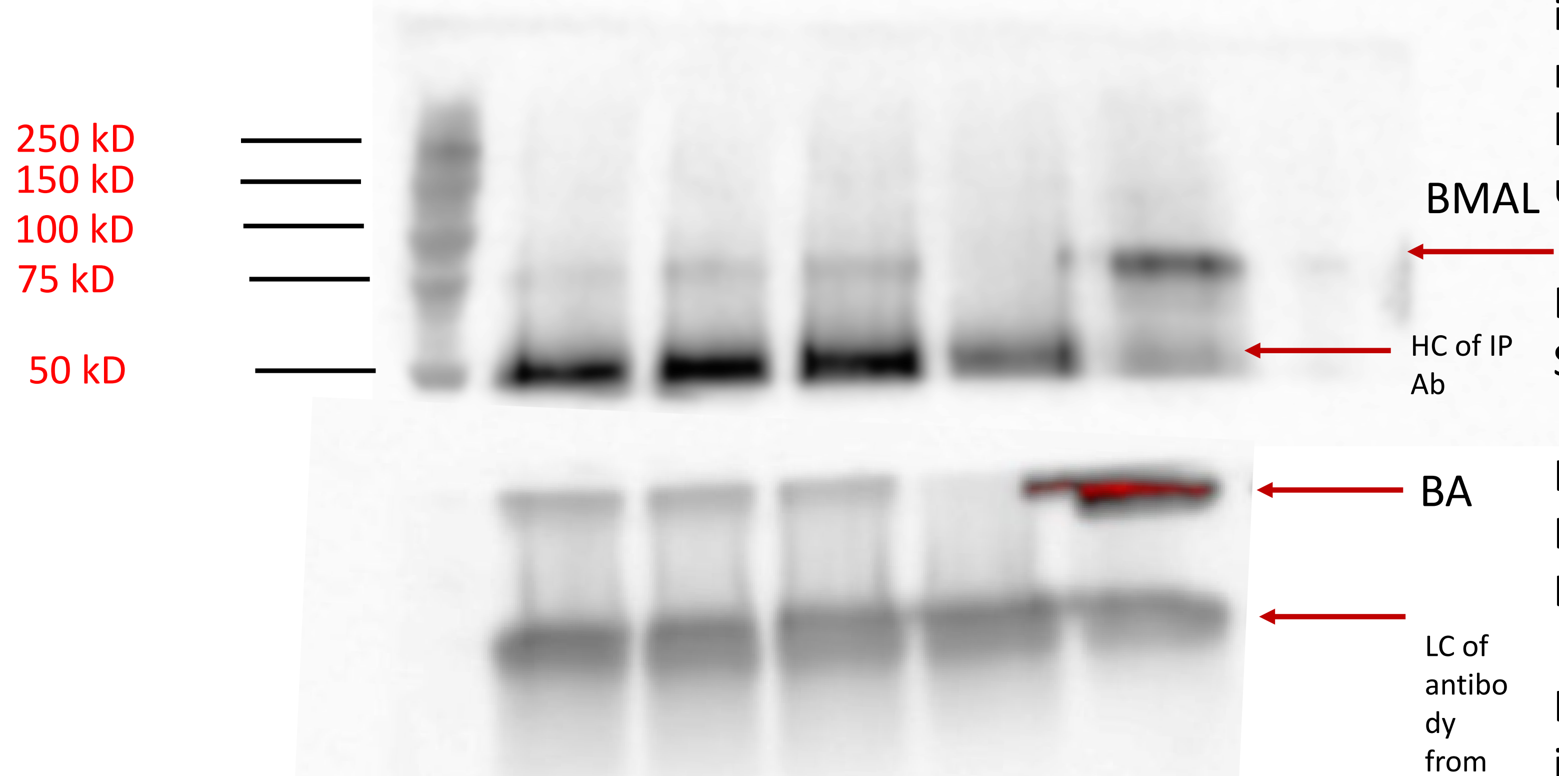

8-24-16 standard IP with HC11 UNDIFF CT12 protein lysate

Lysates were precleared before incubating with Ab (mixed with rotation and Dynabeads G for 1 hr., beads were discarded and lysate was used for experiment

Lane 1 = Precision Plus Protein Standard Ladder

Lane 2= 100 µg
Lane 3= 150 µg
Lane 4 = 200 µg

Lane 5= lysate was precleared and incubated with beads but no Ab (-)

Lane 6= an aliquot of supernatant pulled off of lane 5 sample before wash steps that precede elution

IP : rabbit polyclonal to BMAL 1 (ab3350, ChIP grade, 2 µg per IP)
Western Blot: mouse monoclonal to BMAL 1 (sc-373955 @ 1:750 primary antibody concentration)

Supplemental Figure S2.
Whole gels

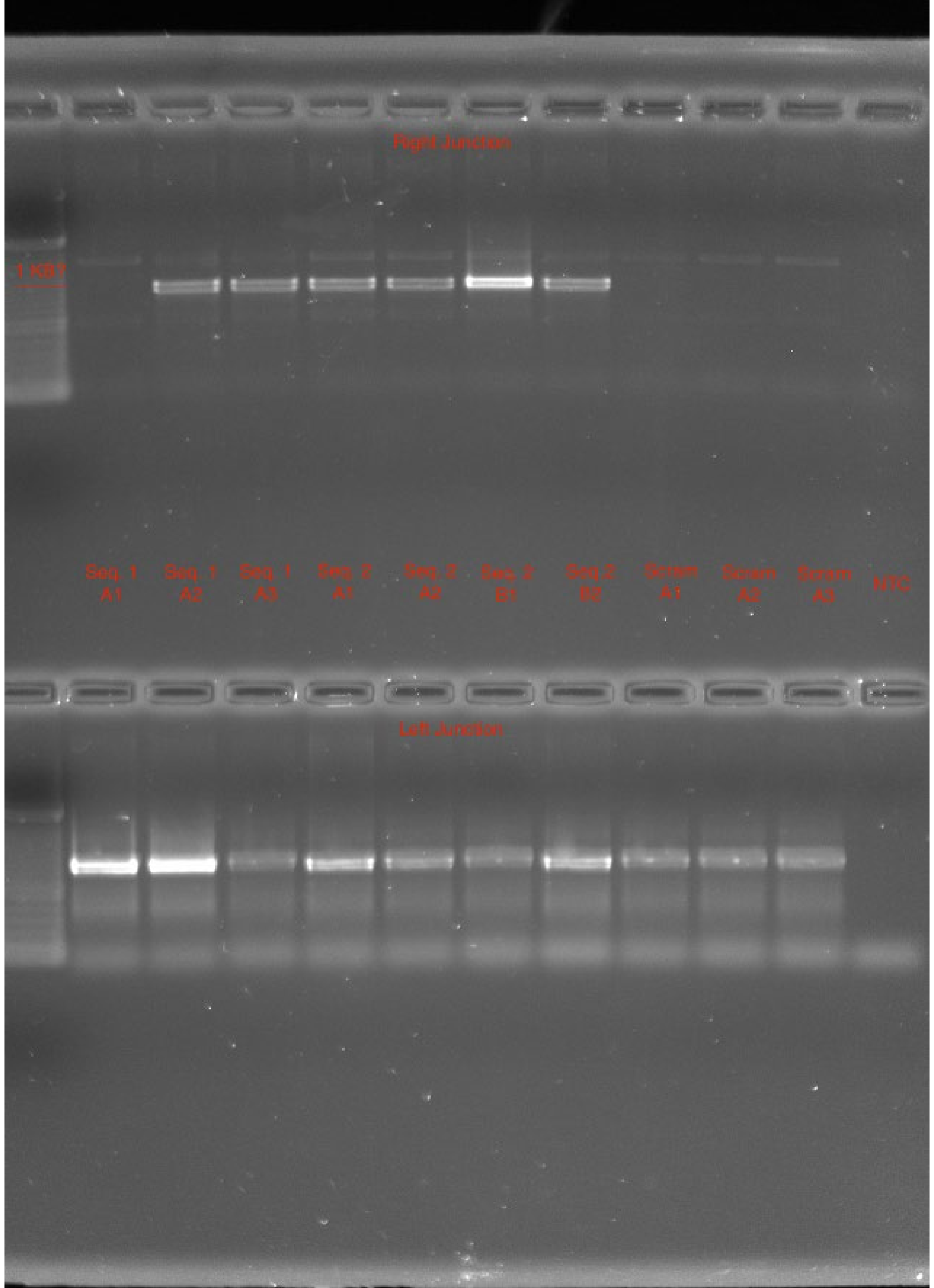

Supplement: S1 Raw images — (PDF) [file pone.0248199.s026.pdf]
